# Seed dimorphism, nutrients and salinity differentially affect seed traits of the desert halophyte *Suaeda aralocaspica* via multiple maternal effects

**DOI:** 10.1186/1471-2229-12-170

**Published:** 2012-09-25

**Authors:** Lei Wang, Jerry M Baskin, Carol C Baskin, J Hans C Cornelissen, Ming Dong, Zhenying Huang

**Affiliations:** 1 State Key Laboratory of Vegetation and Environmental Change, Institute of Botany, Chinese Academy of Sciences, Beijing 100093, China; 2 Department of Biology, University of Kentucky, Lexington, KY, 40506, USA; 3 Department of Plant and Soil Sciences, University of Kentucky, Lexington, KY, 40546, USA; 4 System Ecology, Department of Ecological Science, VU University, De Boelelaan 1085, 1081, HV Amsterdam, The Netherlands; 5 State Key Laboratory of Desert and Oasis Ecology, Xinjiang Institute of Ecology and Geography, Chinese Academy of Sciences, Urumqi, 830011, China

**Keywords:** Bet-hedging, Germination, Seed heteromorphism, Seed morph ratio, Seed size

## Abstract

**Background:**

Maternal effects may influence a range of seed traits simultaneously and are likely to be context-dependent. Disentangling the interactions of plant phenotype and growth environment on various seed traits is important for understanding regeneration and establishment of species in natural environments. Here, we used the seed-dimorphic plant *Suaeda aralocaspica* to test the hypothesis that seed traits are regulated by multiple maternal effects.

**Results:**

Plants grown from brown seeds had a higher brown:black seed ratio than plants from black seeds, and germination percentage of brown seeds was higher than that of black seeds under all conditions tested. However, the coefficient of variation (CV) for size of black seeds was higher than that of brown seeds. Seeds had the smallest CV at low nutrient and high salinity for plants from brown seeds and at low nutrient and low salinity for plants from black seeds. Low levels of nutrients increased size and germinability of black seeds but did not change the seed morph ratio or size and germinability of brown seeds. High levels of salinity decreased seed size but did not change the seed morph ratio. Seeds from high-salinity maternal plants had a higher germination percentage regardless of level of germination salinity.

**Conclusions:**

Our study supports the multiple maternal effects hypothesis. Seed dimorphism, nutrient and salinity interacted in determining a range of seed traits of *S. aralocaspica* via bet-hedging and anticipatory maternal effects. This study highlights the importance of examining different maternal factors and various offspring traits in studies that estimate maternal effects on regeneration.

## Background

Maternal environmental effects occur when the phenotype or growth environment of the mother plant affects the offspring phenotype beyond the direct effect of transmitted genes [1,2]. The seed is simultaneously an important maternal component and a subsequent offspring. Seed traits of the first generation or even of later generations in some annual plants are dependent on the abiotic environment and seed position on the parent plant during seed development and maturation [3]. Seed traits of most species vary between and within individuals, and much of the variation is phenotypic [4]. The adaptive significance of maternal effects on seeds is being increasingly recognized [5-7].

Maternal effects affect a range of offspring traits simultaneously, and these effects are likely to be highly context-dependent [2]. However, most studies of maternal environmental effects on seeds have examined only one maternal factor and one seed trait [4], which may give an incomplete view of the influence of maternal effects [2]. For example, maternal plants of *Senecio vulgaris* grown at lower nutrient levels produced seeds that germinated later and had lower mass than those from plants grown at higher nutrient levels, but their seedlings survived longer when the maternal plant was not supplied with additional nutrients [8]. Thus, investigation of the interactive effect of different maternal factors on variation in seed traits is needed to gain a complete understanding of maternal environmental effects on seed quality, and thereby on the regeneration of populations.

Using an outcome-based approach, Marshall and Uller (2007) distinguished four types of maternal effects: anticipatory (increase in maternal fitness by increasing offspring fitness), selfish (increase in maternal fitness at the expense of offspring fitness), bet-hedging (reduced variation in maternal fitness by producing offspring with a range of phenotypes), and transmissive (reduction in both maternal and offspring fitness). Although this static framework is useful in distinguishing different types of maternal effects, these effects may be entangled.

Seed heteromorphism is a phenomenon in which a single plant produces different morphophysiological types of seeds [9,10]. Seed dimorphism can be considered the main type of this phenomenon, and it is probably a form of bet-hedging that is expressed mostly in annual plants [9,11,12].On the basis of this theory, it is predicted that, in an unpredictable environment, bet-hedging maximizes the geometric mean fitness among generations [13]. However, most studies have focused on changes in the mean offspring phenotype, and only a few studies have been done on variation in offspring phenotypes [13]. In fact, there is variation even within each type of seed in heteromorphic species [14]. To our knowledge, no study has been done on the comparison of seed size variation of heteromorphic species under different environmental regimes. Here we focus on soil fertility and salinity as possible drivers of maternal effects on regeneration.

Nutrient availability to the maternal plant could potentially affect seed production and seed traits [15]. For example, seed production and seed size, germination and other seed quality traits of *Sarcobatus vermiculatus* were decreased substantially with nutrient limitation [16]. This may be a selfish maternal effect, i.e. plants may decrease seed quality and thereby increase total maternal fitness when there is a reduction in the quality of the maternal environment.

Anticipatory maternal effects are predicted to be advantageous in systems where germination conditions are spatially or temporally variable but in a somewhat predictable manner [2]. For example, *Campanula americana* grows in habitats where light is a patchily distributed resource, and their seeds typically experience the same light environment as their mother plant. Germination percentage of this species in autumn was higher when the offspring light environment matched the maternal environment [17]. However, knowledge about the effect of maternal salinity environment on germination of offspring seeds is limited [18]. We expect that seeds produced by maternal plants growing at high salinity would have higher salt tolerance than those produced by mother plants growing at low salinity, because the saline environment is predictable for the offspring of these seeds.

*Suaeda aralocaspica* (Bunge) Freitag & Schütze (Amaranthaceae) is an annual halophyte which is 20–50 cm tall. It occurs in the Gobi Desert of central Asia, where it grows in saline-alkaline sandy soils [19]. This species is a model organism for studying interacting maternal effects on regeneration because it occurs (1) in a heterogeneous environment in terms of soil salinity and fertility; and (2) its seeds are dimorphic [20]. *S. aralocaspica* plants produce brown seeds (non-dormant and high salt tolerance) and black seeds (non-deep physiological dormancy and low salt tolerance) [20]. In this study, we tested the hypothesis that seed dimorphism, nutrients and salinity determine variation in offspring seed traits and germination success of *S. aralocaspica* via the combination of bet-hedging, selfish and anticipatory maternal effects. This main hypothesis leads to the following specific hypotheses:

(1) The seed morph ratio of *S. aralocaspica* can be fine-tuned by plant phenotype (plants from dimorphic seeds) and environmental conditions (salinity and nutrient level). We predict that the brown:black seed ratio should decrease under stressful conditions and therefore be negatively correlated with nutrient limitation and salinity. Additionally, we predict that the brown:black seed morph ratio of plants grown from brown seeds should be higher than that of plants reared from black seeds, because brown seeds germinate faster and these plants may therefore experience better growth conditions during the short window of opportunity for establishment than those from black seeds.

(2) The size variation of brown seeds should be higher than that of black seeds, because brown seeds are dispersed further away from the mother plant than are black seeds; consequently, plants from brown seeds may experience more unpredictable environments. Further, low nutrient and high salinity levels should increase seed size variation.

(3) Maternal nutrient limitation should decrease seed size and germination percentage of *S. aralocaspica*.

## Results

### Seed morph ratio

**Figure 1:**
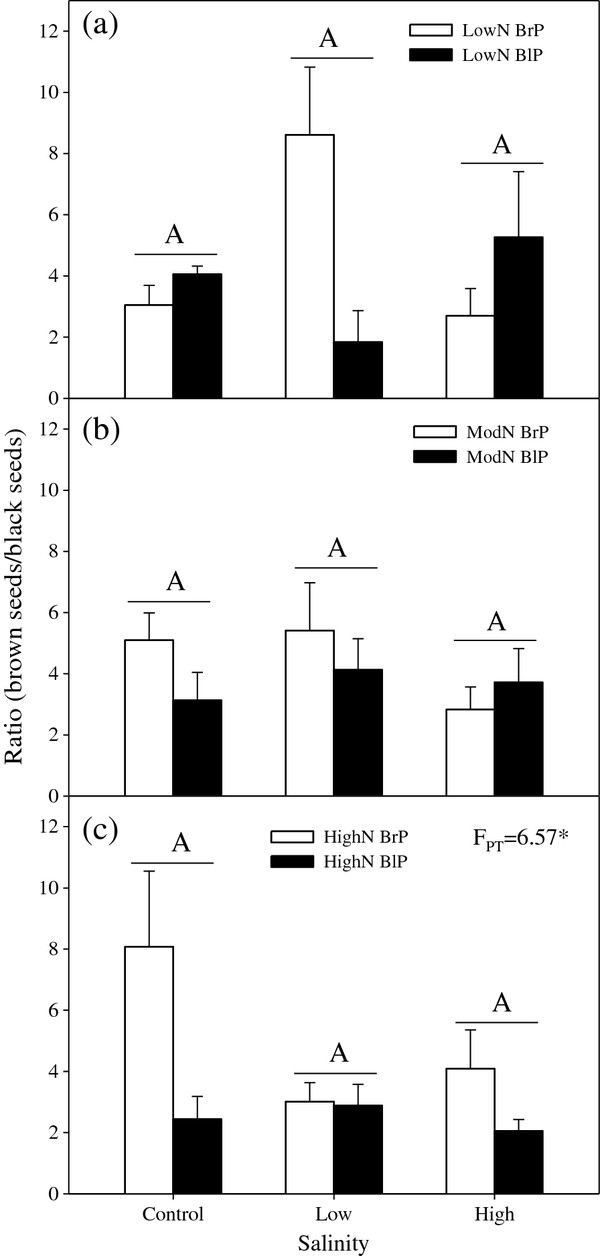
**Effects of plant type and salinity on ratio (number of brown seeds/number of black seeds) per plant for different nutrient availabilities.** (**a**) Low nutrient, (**b**) Moderate nutrient, (**c**) High nutrient. N: Nutrient; BrP: Plant grown from brown seed; BlP: plant grown from black seed. F-values are given when significance levels are reached (PT: plant type; * P < 0.05). Bars with the same uppercase letters are not significantly different at *P <* 0.05 according to the Tukey’s test.

The brown:black seed ratio was significantly affected by plant type (P = 0.028), the interaction between seed type and salinity (P = 0.032) and the interaction among seed type, salinity and nutrient level (P < 0.001) (Table 1). There were no significant differences in the ratio between plants from brown seeds and plants from black seeds under any salt treatment at low or moderate nutrient regimes (Figure 1).

**Table 1:** **Experimental factors and their effect on seed traits of *Suaeda aralocaspica***

**Source of variation**	**df**	**Ratio**		**Seed diameter**			
				**Brown seeds**		**Black seeds**	
		***F***	***P***	***F***	***P***	***F***	***P***
Plant Type (PT)	1	4.997	0.028	0.339	0.561	0.027	0.87
Salinity (S)	2	1.261	0.288	22.411	<0.001	11.295	<0.001
Nutrient (N)	2	0.828	0.44	1.018	0.362	20.347	<0.001
PT*S	2	3.592	0.032	9.18	<0.001	3.669	0.027
PT*N	2	0.777	0.463	1.172	0.311	3.825	0.023
S*N	4	0.296	0.88	2.54	0.04	0.53	0.714
PT*S*N	4	5.624	<0.001	3.371	0.01	2.019	0.091

Results of three-way ANOVA with the following factors: plant type, salinity and nutrient.

Ratio: number of brown seeds/number of black seeds.

In the common garden experiment, the seed morph ratio per plant was 4.09 ± 0.59 in plants from brown seeds and 2.06 ± 0.46 in plants from black seeds (P < 0.001).

### Seed size

**Figure 2:**
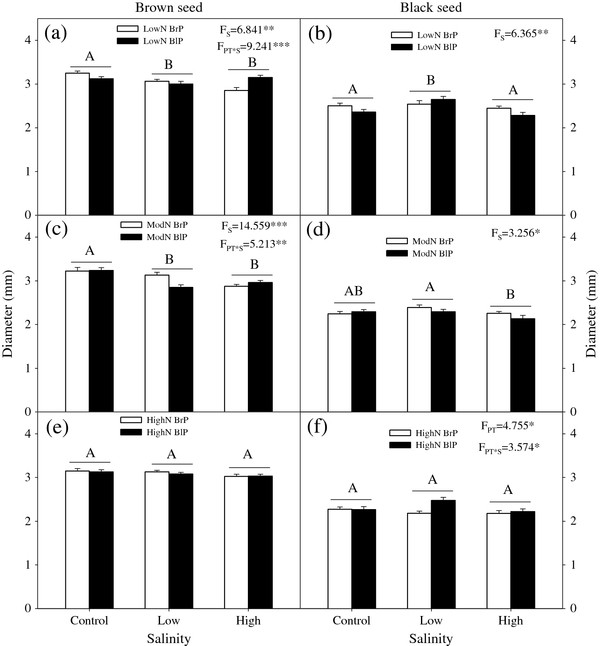
**Effects of plant type and salinity on diameter of brown and black seeds for different nutrient availabilities.** (**a**, **b**) Low nutrient, (**c**, **d**) Moderate nutrient, (**e**, **f**) High nutrient. N: Nutrient; BrP: Plant grown from brown seed; BlP: Plant grown from black seed. F-values are given when significance levels are reached (S: salinity; PT: plant type; * P < 0.05, ** P < 0.01, *** P < 0.001). Bars with the same uppercase letters are not significantly different at *P <* 0.05 according to the Tukey’s test.

There was a significant effect of salinity alone or in combination with plant type or nutrients on diameter of brown seeds. In contrast, there were no effect of plant type or nutrients on diameter of brown seeds and no significant interactive effect between plant type and nutrient regime (Table 1). Salinity significantly decreased the diameter of brown seeds in the low and moderate nutrient regimes, but not in the high nutrient regime (Figure 2).

The main effects of salinity and of nutrients on diameter of black seeds were significant. Additionally, there were significant interactive effects between seed type and salinity (P = 0.027) and between seed type and nutrients (P = 0.023) on diameter of black seeds (Table 1). High nutrients produced the thinnest black seeds. Black seeds in the low salinity treatment were widest in the low and moderate nutrient regimes (Figure 2). Brown seeds were significantly wider than black seeds (P < 0.001) (Figure 2).

### Coefficient of variation (CV) of seed size

**Figure 3:**
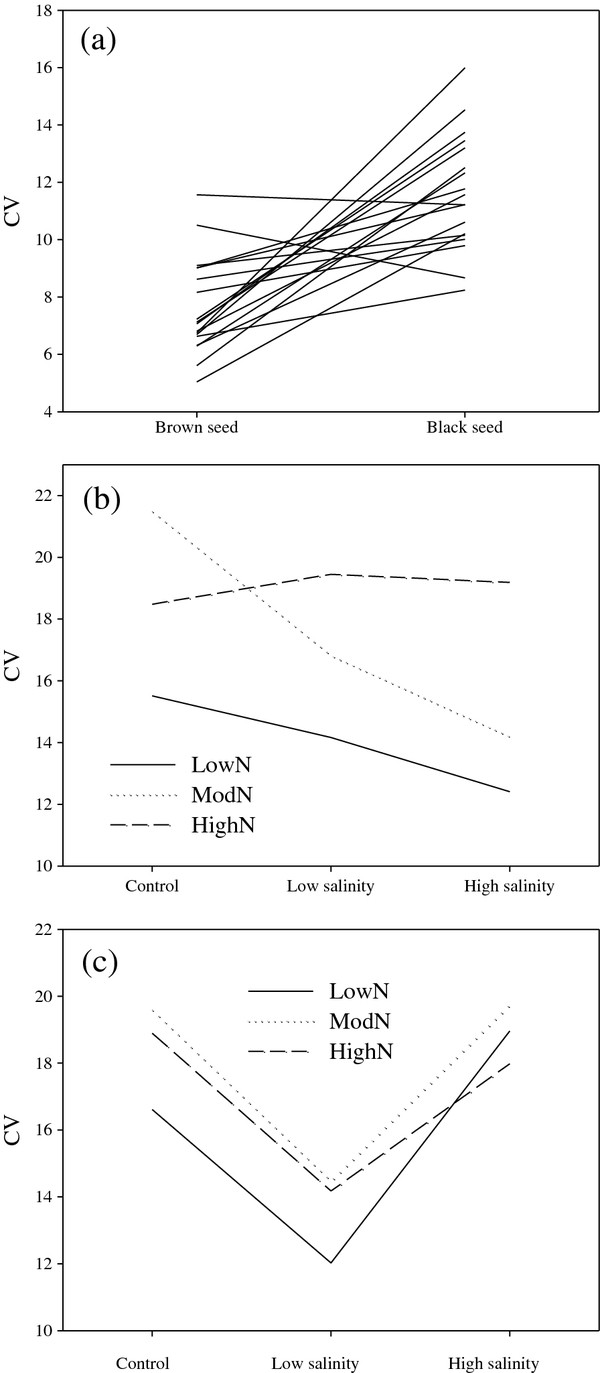
**Reaction norm plots for different drivers of CV (coefficient of variation) of seed diameter.** (**a**) Effect of seed type (brown seed or black seed) on CV in 18 treatments. Each line represents a treatment of *Suaeda aralocaspica*; (**b**) Effect of nutrient (low, moderate and high) and salinity (control, low and high) on CV for plant grown from brown seed and (**c**) for plant grown from black seed. N: Nutrient.

The effects of seed type on CV of seed diameter varied by treatment, with black seeds having a higher CV of diameter in 16 treatments (range 8.24 to 16.00) and brown seeds having a higher CV of diameter in two treatments (range 5.04 to 11.56); the latter under 0 salinity and moderate nutrients and under high salinity and low nutrients (Figure 3a).

Under low maternal nutrient conditions, CV for seed diameter in plants from brown seeds was relatively small, and seeds produced on plants grown at high salinity had the lowest CV for diameter, while those grown at control salinity had the highest CV (Figure 3b). Under moderate maternal nutrients, seeds produced on plants grown at high salinity had the lowest CV for diameter, while those grown at control salinity had the highest CV (Figure 3b). CV for seed diameter in plants from brown seeds under high maternal nutrients was highest when grown under low salinity (Figure 3b).

Under low maternal salinity, CV of seed diameter in plants from black seeds was relatively small, and seeds produced by plants grown in low maternal nutrient conditions had the smallest CV for diameter (Figure 3c). Under moderate maternal nutrient conditions, CV for seed diameter in plants from black seeds was highest (Figure 3c).

### Seed germination

**Figure 4:**
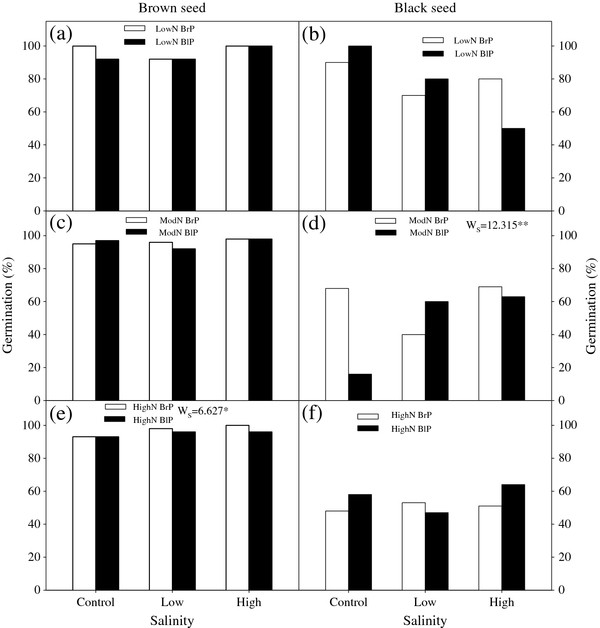
**Effects of plant type and maternal salinity on germination of brown and black seeds for different nutrient availabilities.** (**a**, **b**) Low nutrient, (**c**, **d**) Moderate nutrient, (**e**, **f**) High nutrient. N: Nutrient; BrP: Plant grown from brown seed; BlP: Plant grown from black seed. W-values are given when significance levels are reached (S: salinity; * P < 0.05, ** P < 0.01).

Germination of *S. aralocaspica* seeds was significantly affected by maternal soil nutrient levels, salinity and seed morph but not by type of mother plant (Figure 4). Germination percentage of brown seeds was higher than that of black seeds under all treatments. Maternal nutrient level did not affect germination percentage of brown seeds. For black seeds, higher maternal nutrient levels significantly decreased germination percentage (P = 0.005). High maternal salinity significantly increased germination percentage of brown seeds for high maternal nutrient level (P = 0.036) and of black seeds for moderate maternal nutrient level (P = 0.002).

**Figure 5:**
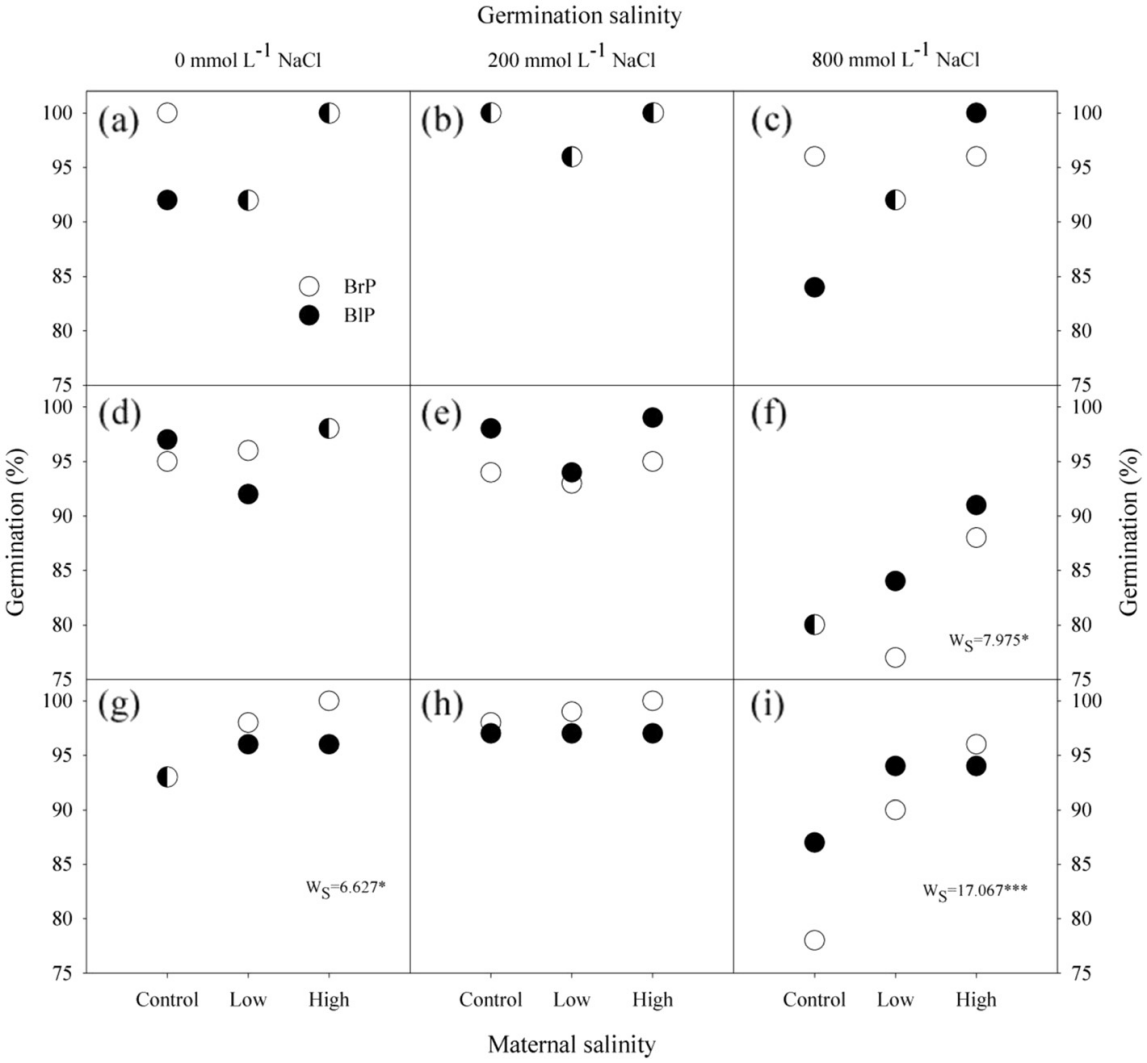
**Effects of plant type and salinity on final germination percentages of brown seed cultivated from different gradients of nutrients and salt concentrations.** (**a**, **b**, **c**) Low nutrient, (**d**, **e**, **f**) Moderate nutrient, (**g**, **h**, **i**) High nutrient. N: Nutrient; BrP: Plant grown from brown seed; BlP: Plant grown from black seed. W-values are given when significance levels are reached (S: salinity; * P < 0.05, *** P < 0.001). Over-lapping values are represented split white-black data points.

When brown seeds were germinated in 0 and 200 mmol L^-1^ NaCl solution, maternal salinity did not affect germination, except for brown seeds from plants incubated at high maternal nutrient level. When brown seeds were germinated in 800 mmol L^-1^ NaCl solution, high maternal salinity significantly increased final germination percentages for moderate (P = 0.019) and high (P < 0.001) maternal nutrient levels (Figure 5). However, seed viability was not affected by salt concentration and the percentage of total viable seed was 100.

## Discussion

Our study of maternal environment effects on seed traits in the *S. aralocaspica* produced four main results. (1) Plants reared from brown seeds had a higher brown:black seed ratio than those reared from black seeds, and soil nutrient and salinity levels did not significantly affect seed morph ratio. (2) Seed dimorphism, nutrient and salinity regulated seed size variation. (3) Maternal nutrient limitation significantly increased seed size and germination percentage of black but not brown seeds. (4) Seeds produced by plants growing at high salinity had higher salt tolerance than those from low salinity plants. The first and second results support bet-hedging maternal effects. The third result did not support selfish maternal effects, but it did support anticipatory maternal effects. The fourth result also supports anticipatory maternal effects.

### Bet-hedging

Seed dimorphism may be a typical example of diversified bet-hedging, where offspring produced by each plant has two distinct ecological strategies to cope with variable environments. Our study revealed that the brown:black seed ratio of *S. aralocaspica* is not controlled by variable maternal environmental conditions but by plant type (plants grown from brown or black seeds) (Figure 1). However, environmentally-altered ratios of seed morphs previously have been reported [6,14,21,22]. A consistent trend for the ratio (propagules with high dispersal)/(propagules with low dispersal) has been found in amphicarpic plants [23-25]. Thus, under stressful conditions plants tend to produce relatively more subterranean seeds with low dispersibility, and under very stressful conditions no aerial seeds are produced [24]. For heteromorphic plants, stressful conditions may increase [21,26], decrease [25,27-29] or not alter [30,31] the seed morph ratio. Plants of the amphicarpic species *Emex spinosa*[32], *Cardamine chenopodifolia*[33] and *Catananche lutea*[25] from subterranean seeds produced more aerial fruits and seeds and allocated more biomass to aerial reproduction than those from aerial seeds.

A decrease under stress of the ratio (propagules with high dispersal)/(propagules with low dispersal) in amphicarpic plants has not been found in most heterodiasporous plants. Plants reared from black seeds of *Chenopodium album* (Amaranthaceae) produced more brown seeds than plants from brown seeds, but this phenomenon was only observed for plants reared at 50 mmol L^-1^ NaCl [22]. Plants reared from heteromorphic diaspores of *Dimorphotheca sinuata* (Asteraceae) and *Arctotis fastuosa* (Asteraceae) did not differ in ratio of seed morphs [34]. Our results indicated that plants grown from brown seeds produced a higher brown:black seed ratio than those produced from plants grown from black seeds in all nutrient and salinity treatments, which was confirmed by the common garden experiment. This pattern can be considered as a new model of heteromorphic species behavior. That is, the seed morph ratio of *S. aralocaspica* remains constant despite changes in maternal nutrient supply and salinity. The influence of plant type on seed morph ratio may have important consequences for ecological adaptation. Brown seeds have high germinability and dispersability and represent the high risk strategy [9,20], whereas black seeds have low germinability and dispersability and represent the low risk strategy [9,20]. Thus, the brown:black seed ratio may represent the ratio of (high dispersal/nondormancy)/(low dispersal/high dormancy), a risk index. Given that plants grown from brown seeds have a higher risk index, it is expected that most plants in new habitats came from brown seeds. Meanwhile, plants reared from black seeds have a lower risk index and remain in the maternal habitats, especially those that are frequently disturbed. Hence, our results suggest plastic bet-hedging in the halophyte *S. aralocaspica* that allows plants to cope with unpredictable environmental variability.

### Dynamic bet-hedging

Theory suggests that mothers should increase within-clutch variation in the offspring phenotype when they can not predict their offspring’s environmental conditions [13]. In *S. aralocaspica,* size variation of black seeds is higher than that of brown seeds (Figure 3). Seed dispersal is often limited, and most seeds of plants are dispersed relatively close to the maternal plant [35,36]. Primary (gravity-driven) and secondary (over the ground by wind) seed dispersal of *S. aralocaspica* is limited. Most brown and black seeds are likely to remain in their mother’s habitat, though dispersal distance of brown seeds is greater than that of black seeds (personal observation). All brown seeds germinate the next spring following maturity in autumn. Only a portion of the black seeds, which have non-deep physiological dormancy, can germinate in their first spring, and the others become part of a persistent soil seed bank [20]. Therefore, compared to brown seeds black seeds experience more unpredictable temporal environments and have higher variation in seed size.

Plants of *S. aralocaspica* grown from brown seeds produced seeds with lower size variation under low levels of nutrient and high salinity, and plants grown from black seeds produced seeds with lower size variation under low levels of nutrients and salinity (Figure 3). Thus, low nutrient and high salinity are predictable environmental conditions for plants grown from brown seeds, and low levels of nutrients and salinity are predictable environmental conditions for plants grown from black seeds. In salt deserts, low nutrient levels are predictable, and seeds produced by plants of *S. aralocaspica* from brown and black seeds had the lowest CV under low nutrient. Brown seeds can germinate at high salinity, and seedlings from brown seeds can survive at high salinity. Thus, the high maternal salinity is predictable for plants from brown seeds. However, black seeds germinate at low salinity, and seedlings from black seeds survive at low salinity. Hence the low maternal salinity is predictable for plants from black seeds. Seed size variation in response to changes in environmental predictability may be viewed as dynamic bet-hedging, an adaptive maternal effect [13]. Our study is the first to demonstrate variation of seed size of each seed morph as affected by specific maternal environment.

### Anticipatory maternal effects

While maternal nutrient limitation simply reduces the number of seeds, its impact on seed size and germination is more complex. Nutrient limitation is reported to decrease, to increase or not to change seed size and germination percentage [14,15]. The most striking finding of our study was that maternal nutrient limitation increased size of black seeds but had no significant effect on size of brown seeds in *S. aralocaspica* (Table 1; Figure 2). The physiological mechanism of this phenomenon needs to be explored. Varying environmental conditions may cause changes not only in seed size but also in germination percentage. Changes in seed size may be related to seed germination; in many cases large seeds germinate better than small ones [37]. Our black seeds produced under low levels of nutrients had the widest diameter and the highest germination percentage. A large store of resources allows black seeds to germinate successfully under low nutrient conditions, thereby enhancing offspring fitness in *S. aralocaspica*. Under stressful conditions, maternal fitness is increased by increasing offspring fitness. In mother plants, size and germination of black seeds is adjusted according to local nutrient conditions and thus maximize offspring and maternal fitness. This phenotypic plasticity is the anticipatory maternal effect also found in other plants [2].

Maternal environment salinity had strong effects on seed germination in *S. aralocaspica*. Seeds produced by plants grown at high maternal salinity had higher germination percentages than those produced by plants grown in low maternal salinity regardless of germination salinity (Figure 4; Figure 5). Previous work demonstrated that seeds from maternal plants grown at high salinity germinated more readily at high salinity than seeds from low-salinity plants [38]. The salinity environment of maternal plants of *Atriplex nummularia* profoundly affected the germination response of their seeds [39]. *Iris hexagona* seeds from high-salinity mothers germinated earlier than those from low-salinity mothers, but these effects were not detectable 10 days after germination [18]. Maternal effects that have a significant influence on plant fitness may confer a fitness advantage in environmental conditions similar to those experienced by the parents [15,40]. The pattern of germination percentage in *S. aralocaspica* suggests that maternal effects are more strongly expressed at high than at low salinity. We suspect that this kind of maternal effect is an anticipatory maternal effect [2].

## Conclusions

We have documented multiple maternal effects on seed traits of *S. aralocaspica*. As predicted, seed morph ratio and seed size variation fine-tuned by maternal factors are bet-hedging maternal effects. Increase in salt tolerance of offspring seeds through the effects of maternal salinity are anticipatory maternal effects. Increase in seed size and in germination percentage through maternal nutrient limitation is also an anticipatory as opposed to selfish maternal effect. Multiple interacting aspects of maternal plant phenotype and maternal environments together determine various seed traits via different maternal effects. Our findings add to the increasing evidence that plants have evolved multiple maternal mechanisms that, together, may help to optimize long-term regeneration and maintenance of populations in environments with varying and unpredictable levels of environmental stress.

## Methods

### Study species

In China, *S. aralocaspica* is found only in the inland cold desert of the Junggar Basin, Xinjiang Province. Plants bloom in August and produce two types of seeds on the same plant in September that differ both morphologically and ecologically [20]. One seed type is oblate and brown with a soft seed coat. It is about 3.2 mm in diameter, non-dormant, has a high level of germinability (when fresh) and can be dispersed relatively long distance by wind. The other seed type is elliptical and black with a rigid seed coat. It is about 2.5 mm in diameter, dormant, has a low level of germinability (when fresh) and can be dispersed only a relatively short distance by wind [19,20].

### Seed collection

Freshly matured fruits of *S. aralocaspica* were collected from plants in a natural population (44^o^14' N; 87^o^44' E; 445 m a.s.l) growing in saline desert soils near the Fukang Desert Ecosystem Observation and Experimental Station (FDEOES), Xinjiang Institute of Ecology and Geography, Chinese Academy of Sciences, in Xinjiang Province, China, on 5 October 2008. Fruits were taken from at least 200 plants and allowed to dry naturally for 10 days at ambient room conditions. Seeds were separated from the dried plant material and sorted into brown and black seeds. Then, each type of seed was pooled, thoroughly mixed and stored dry at 4°C in a closed cotton bag until used in experiments.

Salinity of soil samples collected from 12 randomly chosen sites within the study population in October, 2008 were analyzed by the residue drying quality measure [41]. Mean total soil salinities in the 0-20-cm soil layers were 5.10 ± 0.62% (873 ± 106 mmol L^-1^ NaCl). Minimum and maximum soil salinities were 1.95% (334 mmol L^-1^ NaCl) and 8.64% (1478 mmol L^-1^ NaCl), respectively.

Soil nutrients in patches of *S. aralocaspica* natural habitats were also analyzed by standard methods [41]. Mean contents of total nitrogen (N), phosphorus (P) and potassium (K) were 0.48 ± 0.02, 0.72 ± 0.02 and 18.44 ± 0.41 g kg^-1^, respectively, and available N, P and K 64.23 ± 4.02, 7.86 ± 1.31 and 235.10 ± 13.98 mg kg^-1^, respectively.

### Experimental design

#### ***Experiment 1: pot experiment***

The experiment was carried out in a well-ventilated screen house at FDEOES (44^o^17′26″N, 87^o^55′58″E; 460 m a.s.l.), located in the southern part of the Junggar Basin of Xinjiang Province, China. During the experiment, the screen house was covered with shade net (ca. 25% porosity) and also with plastic cloth during rains.

Twenty to thirty seeds of each of the two morphs were sown into experimental pots 17 cm deep and 16 cm in diameter filled with 2000 cm^3^ of a vermiculite quartz-sand mix (4:1 v/v) on 27 April 2009. The sowing date was concurrent with the emergence of *S. aralocaspica* seedlings at the Fukang site in 2009. Seedlings were grown for 20 days and then thinned to only one per pot. To reduce variation in initial seedling size, only seedlings of the same height were used in the experiment.

A randomized block design with twelve replicates was used. Each block consisted of 18 pots representing a combination of the two seed morphs (brown and black), three fertilization treatments (low, moderate and high) and three salinity levels (0 as control, low and high). There were a total of 216 plants in this experiment. The levels of nutrient and salinity matched the soil characteristics of the habitat of *S. aralocaspica*. For the fertilization treatment, a commonly available granular lawn fertilizer (Osmocote 301, Scotts, Marysville, OH, USA) with a 15 N : 11P : 13 K : 2 Mg elemental ratio was used as the basic fertilizer and a commercial nutrient solution (Peters1, Scotts) with a 20 N : 20P : 20 K elemental ratio as the supplemental fertilizer. In the fertilization treatment, each pot received (1) 1.2 g Osmocote 301 once and 100 mL Peters1 nutrient solution (0.046 g L^-1^) once a week (low fertilization), (2) 6 g Osmocote 301 once and 100 mL Peters1 nutrient solution (0.23 g L^-1^) once a week (moderate fertilization) or (3) 12 g Osmocote 301 once and 100 mL Peters1 nutrient solution (0.46 g L^-1^) once a week (high fertilization). Addition of the Peters1 nutrient solution began 3 weeks after sowing. For the salinity treatment, a mixed salt with a 20NaCl : 20Na_2_SO_4_ : 1NaHCO_3_ (mass ratio) was used. Each pot received (1) 100 mL tapwater (0 g L^-1^) (control) once a week, (2) 100 mL salt solution (1 g L^-1^) once a week or (3) 100 mL salt solution (7 g L^-1^) once a week. To avoid osmotic shock, 7 g L^-1^ salinity was applied gradually by adding 1 g L^-1^ salinity per week. Adding the salt solution started 4 weeks after sowing. Pots were watered every 2 days, and the same amount of water/ nutrient solution/ salt solution was used for each pot.

There were 12 replicates in each treatment. Some mortality occurred, and the final sample sizes were reduced to 8–11 replicates for different treatments. We randomly chose six replicates in each treatment at the end of the experiment. Over the 160 day period of growth, average (daily) maximum and minimum temperatures were 30.2°C (range 26.2-33.7°C) and 12.6°C (range 9.0-16.7°C), respectively. Average (daily) maximum relative humidity (RH) was 39%, and the average (daily) minimum RH was 18%. Maximum illuminance occurred at about 2 pm, and average (daily) maximum illuminance in the screen house was 2.5 × 10^4^ lux, which is 25% of full sun illuminance at midday on a clear day in summer. We harvested the plants of *S. aralocaspica* at the end of the growing season and measured seed number, seed size and germination percentage.

#### ***Experiment 2: common garden experiment***

This experiment was done to verify (or not) the seed morph ratio pattern in the first experiment. On 22 April 2009, the common garden at FDEOES was watered to field capacity. On 24 April 2009, ca. 2000 seeds of each morph were sown in the common garden. One 2.5 × 10 m plot of each morph was surrounded by a common dike so that the same water would flood both plots to the same depth when they were irrigated. Plants were watered once every 2 months using the same amount of water as in the first irrigation. No fertilizer was applied to the plots. In August, 18 black-seeded plants and 70 brown-seeded plants remained. Brown-seeded plants were thinned to 30 individuals to make sure they had enough space to grow without competition. We harvested the plants at the end of their growing season and determined seed morph ratio (see below) of ten random individuals per plot.

Salinity and nutrient concentrations of soil samples collected from 4 randomly chosen sites within the common garden on 20 October, 2009 were analyzed as in experiment 1. Mean total soil salinities in the 0-20-cm soil layers were 1.61 ± 0.11% (276 ± 19 mmol L^-1^ NaCl). Mean contents of total nitrogen (N), phosphorus (P) and potassium (K) were 0.76 ± 0.06, 1.16 ± 0.09 and 14.10 ± 0.67 g kg^-1^, respectively, and available N, P and K 96.23 ± 13.42, 9.29 ± 3.52 and 534.33 ± 36.39 mg kg^-1^, respectively.

### Seed traits

The number of each seed morph for each individual plant was determined. Seed morph ratio is total number of brown seeds divided by total number of black seeds on a single plant. The diameters of twenty randomly selected seeds of each morph from each combined treatment were measured using a computer imaging system. Seed size variation was expressed as coefficient of variation per treatment (CV = standard deviation/mean).

### Germination experiments

#### ***Effects of cultivation treatments on germination***

Freshly harvested seeds were stored at laboratory conditions (21–25°C, 40–45% relative humidity) for two weeks before germination was tested. To investigate the germination behavior of offspring seeds, four replicates of 25 fresh seeds of each morph produced by plants in each combined treatment were incubated on two layers of Whatman No.1 filter paper moistened with 2.5 mL of distilled water in 5-cm-diameter plastic Petri dishes. Petri dishes were placed in closed plastic bags and incubated at a daily (12:12-h) temperature regime of 10:25°C (mean daily maximum and minimum temperatures at the FDEOES in late April and May) in light (12-h daily photoperiod, fluorescent lamp, 1.2 × 10^4^ lux) for 20 d. A seed was considered to have germinated when the radicle had emerged > 0.5 cm. Germination was examined every 48 h, and seedlings were removed at each count.

#### ***Effect of salinity on germination***

The effects of 0 (distilled water control), 200 and 800 mmol L^-1^ NaCl on germination of brown seeds produced by plants in each combined treatment were tested at a daily temperature regime of 10:25°C in light (12-h daily photoperiod). There were four replicates of 25 seeds in 5-cm-diameter plastic Petri dishes. Seed germination percentages were calculated after incubation for 20 d. Viability of seeds that did not germinate in the experiments was tested by the TTC method (embryos are placed in a 1% solution of 2,3,5-triphenyl-2 H-tetrazolium chloride) and those that turn pink are viable [37].

### Statistical analysis

All analyses were performed with SPSS Version 13.0 (SPSS, Inc., Chicago, IL, USA), and data were log-transformed where necessary to improve normality and homogeneity of variances. We used three-way ANOVA to determine the effects of plant type, salinity and nutrients and their interactions on brown:black seed ratio, diameter of brown seeds and diameter of black seeds. Tukey’s HSD test and paired two-tailed tests were performed for multiple comparisons to determine significant (P < 0.05) differences between individual treatments.

Germination data were analyzed using binary logistic regression. The logistic model included cultivation salinity (0 as control, low and high), plant type (brown-seeded plant and black-seeded plant) and their interaction. The significance of the factors and interactions in the selected model was tested by Wald χ^2^ values [42].

## Competing interests

The authors declare that they have no competing interests.

## Authors’ contributions

JB, CB and ZH designed the study; LW and ZH performed the experiments; LW, ZH and MD analyzed the data; and LW, CB, ZH and JC wrote the manuscript, which was further edited by JB and MD. All authors read and approved the final manuscript.
